# Brain activation during cognitive control tasks differs substantially between people but is reliable within individuals

**DOI:** 10.1162/IMAG.a.995

**Published:** 2025-11-10

**Authors:** Katherine J. Michon, Quan Zhou, Jahla B. Osborne, Adriene M. Beltz, John Jonides, Molly Simmonite, Thad Polk

**Affiliations:** Department of Psychology, University of Michigan, Ann Arbor, MI, United States; Department of Psychiatry, University of Michigan, Ann Arbor, MI, United States

**Keywords:** precision neuroimaging, cognitive control, fMRI, reliability

## Abstract

The neural organization of cognitive control has been extensively studied using neuroimaging methods, but this organization is still not well understood. We argue that two factors may have contributed to this elusiveness. First, most previous research has relied on group-averaged results, which may provide a misleading representation of individual brains. Second, most fMRI studies study the brain only under a limited number of conditions, making it challenging to provide fine-grained distinctions in the functions associated with specific regions. Recent precision neuroimaging approaches have demonstrated substantial promise in furthering understanding of the human brain through repeated sampling of individual participants. However, most precision imaging work still relies on resting-state fMRI or a small number of tasks. In the present study, we demonstrate the utility of a novel dense imaging approach, which combines precision neuroimaging with an unusually large task battery. We demonstrate that patterns of neural activity associated with cognitive control tasks are significantly more similar within-person than between people, even after controlling for anatomical similarity, suggesting that these patterns are person-specific and reliable. In addition, we demonstrate that within-person and between-person similarity changes significantly across tasks, suggesting that some tasks may be more suited for exploring individual differences in cognitive control than others. Together, our findings highlight the potential value of a precision approach and the benefit of using a large number of tasks to further understanding of cognitive control.

## 1 Introduction

Cognitive control is the ability to flexibly act on internally represented goals and is essential to human behavior (Miller & Cohen, 2001). Cognitive control (also called ‘executive control’, ‘executive function’, or more simply, ‘control’) also varies substantially across people, and while better control abilities are linked with a wealth of positive health outcomes, impairments in control are linked to a wide variety of psychiatric and neurological conditions (Bailey, 2007; Blair & Razza, 2007; Borella et al., 2010; Braver & Barch, 2002; Chen et al., 2019; Chuderski & Necka, 2010; Diamond, 2013; Hakun & Findeison, 2020; Llewellyn et al., 2008; Shields et al., 2016). Decades of neuroimaging research have been dedicated to elucidating the neural underpinnings of cognitive control, but there is still no clear consensus on its neural organization, which also includes substantial interindividual variation (Badre & Nee, 2018; C. Gratton, Sun, et al., 2018; G. Gratton et al., 2018; Mueller et al., 2013; Peña-Gómez et al., 2018; Smith et al., 2021).

Although traditional approaches to neuroimaging research have led to important advancements in the understanding of cognitive control, they also have some important limitations that may hinder that understanding. For example, most functional MRI (fMRI) research relies on group-averaged results and the assumption that functional areas have the same spatial organization across individuals. However, studies in mathematics, psychology, and neuroscience have shown that group-averaged results almost never truly reflect the individuals from which the average was drawn (Beltz et al., 2016; Molenaar, 2004; Seghier & Price, 2018). A second characteristic of most traditional fMRI studies is that they collect data only during a limited number of contexts relevant to a specific question. However, if the goal is to characterize the function of specific brain areas in detail, it would be helpful to observe activity (or the lack of activity) during a wide range of contexts. For example, a great deal has been learned about the function of areas in the ventral visual cortex (e.g., the fusiform face area, the parahippocampal place area, etc.) by observing activity in these regions while participants view a very wide range of visual stimuli and determining which stimuli activate a given region and which stimuli do *not* activate it (Busch et al., 2021; Haxby et al., 2011; Kanwisher et al., 1997).

Novel fMRI approaches that aim to address such limitations may provide further insight into the neural organization of cognitive control. One such approach, called precision neuroimaging, has demonstrated substantial promise in elucidating details of the brain’s neural organization (Braga & Buckner, 2017; DiNicola et al., 2020; Gordon et al., 2017, 2020; Gordon & Nelson, 2021; C. Gratton, Laumann, et al., 2018; Laumann et al., 2015; Lynch et al., 2019; Poldrack et al., 2015; Seitzman et al., 2019). Whereas most traditional neuroimaging studies collect relatively small amounts of data from each person, precision neuroimaging studies collect several hours of data per individual (Gratton et al., 2020; Michon et al., 2022). By obtaining high-quality individual measurements, precision neuroimaging studies have revealed previously unseen details of the brain’s neural architecture.

For example, Braga and Buckner (2017) investigated the neural organization of 12 individuals scanned over several hours each and compared that neural organization to large-scale networks based on group-averaged results. They found that the default mode network, the frontoparietal network, and the dorsal attention network fractionated into multiple separate networks at the individual level, confirming that group-averaged results might obscure important details of neural organization. A later analysis of 10 highly sampled participants found nine different subnetworks in the default mode network (Gordon et al., 2020). In both cases, these results would not have been found if not for the detailed, person-specific data and analysis. In fact, it seems that individual-specific features that are not present in group-averaged results may exist in many people, and these features may have some relevance to behavior. Individuals assigned to different feature subgroups demonstrated differences on neuropsychological behavioral measures, including life satisfaction and drug abuse (Gordon & Nelson, 2021; Seitzman et al., 2019). If these individual differences are relevant to behavior, then gaining a complete and detailed understanding of the human brain might be hindered by relying exclusively on group-averaged neuroimaging results.

Most precision neuroimaging work thus far has relied on resting-state fMRI, which, while useful, may not be optimal for the study of cognitive function (Finn, 2021; Finn et al., 2017; Michon et al., 2022). Instead, some researchers have suggested that a true characterization of cognitive processes requires studying the brain during many different contexts. In this way, experimental tasks may function as a “stress-test”, bringing out changes in the brain’s neural organization that are not typically seen during rest (Finn et al., 2017). In an exploratory analysis, Finn et al. (2017) measured within- and between-person variability in functional connectivity profiles using tasks from the Human Connectome Project. They found that while individuals’ connectivity profiles were more similar to each other during tasks than during rest, the tasks themselves also differed from each other in both within- and between-person variability. The authors suggest that consideration of such information could improve the ability to relate individual differences in neural measures to individual differences in behavior (e.g., by using tasks that exhibit reliable between-person differences while also capturing features that are reliable within-person) (Finn et al., 2017). However, this study used the seven tasks from the Human connectome project, spanning a variety of cognitive functions. More so, these findings concern the variability of connectivity profiles, rather than spatial variability (i.e., interindividual variability in patterns of neural activity across the brain). It remains to be seen whether this difference would extend to spatial variability, especially when using a larger battery of tasks, or if such differences would be found among tasks measuring the same cognitive function (e.g., cognitive control).

Given these arguments, some researchers have suggested that the true function of a specific brain region can only be determined after having observed that brain region’s activity during a wide variety of task contexts (Thirion et al., 2021). In other words, this type of *dense* sampling of individuals as they participate in a large number of tasks could lead to an in-depth characterization of brain function, and because of the same large-scale similarities that are present in the brain, this characterization might generalize to many people (Naselaris et al., 2021).

The present study explores the utility of a novel neuroimaging approach that combines precision neuroimaging with an unusually large task battery. We collected approximately 12 hours of data from two individuals while they performed 22 tasks related to different aspects of cognitive control. We use this novel dataset to expand on previous precision neuroimaging work and demonstrate that patterns of neural activity during cognitive control tasks (measured via fMRI contrast data) differ substantially between people (i.e., they are person-specific), and are largely reliable within people, much like patterns of functional connectivity found in previous studies (Gordon et al., 2017; Laumann et al., 2015). We also show that both within-person and between-person similarity differs significantly depending on the task, which has important implications for research aimed at relating individual differences in neural activity to individual differences in behavior (Finn et al., 2017).

## 2 Methods

### 2.1 Mapping control densely (MCD) dataset

#### 2.1.1 Participant characteristics

Data were collected from two healthy, right-handed female participants, aged 32 and 25 at the beginning of scanning. Both participants were screened for contraindications for MRI and provided informed consent. The study was approved by both the University of Michigan Health Sciences and Behavioral Sciences Institutional Review Board (IRB-HSBS; for experimental tasks) and the Institutional Review Board of the University of Michigan Medical School (IRBMED; for MR procedures). We also provide an analysis of 55 participants from the Dual Mechanisms of Cognitive Control dataset (DMCC55B, with one baseline session of four cognitive control tasks) to demonstrate that these two participants are not more different than would be expected in a larger sample (see section 2.2.2.2.1) (Etzel et al., 2022).

#### 2.1.2 Data acquisition

Imaging data were collected on a GE MR750 3T MRI scanner at the University of Michigan fMRI Laboratory. Participants’ heads were stabilized using foam padding to reduce movement and ensure participant comfort. A Celeritas 5-button fiber optic response system was attached via Velcro to participants’ hands to record responses during task runs. Participants completed 8-9 scanning sessions on separate days, each lasting 30–90 minutes. The scanning sessions included the collection of T1-weighted high-resolution anatomical images using spoiled 3D gradient-echo acquisition (FOV = 220 mm, voxel size = 1 x 1 x 1 mm, 156 slices, TR = 11.42 ms, TE = 5.05 ms, inversion time = 500 ms, flip angle = 15°) and functional images using a single-shot gradient-echo (GRE) reverse spiral pulse sequence (FOV = 220 mm, voxel size = 3.4 x 3.4 x 3 mm, 43 slices, TR = 2000 ms, TE = 30 ms). Automated shimming was applied as part of standard procedures to minimize field inhomogeneities during the scans.

#### 2.1.3 fMRI task design

During each scanning session, participants completed a subset of 22 cognitive control tasks in a test-retest fashion, such that each task was completed twice on separate days. The order of the tasks can be found in Table 1. All of the tasks were adapted from previously published studies and created to be block design; however, two tasks (the Inhibit, Switch, Update task and the Go/No-Go task) were analyzed as event-related based on their associated publications. The task order was identical across participants and test/retest sessions to avoid differences in brain activation due to task order. During the first scanning session, participants completed one run each of the first nine tasks. These same nine tasks were completed again in the second session. Sessions 3 and 4 consisted of 4–5 runs of a single task (see Table 1; one participant completed only 4 runs during session 3 due to illness). In sessions 5 and 6, participants completed one run each of 8 tasks, and the last 4 tasks were completed during session 7 and again in session 8. Before completing each new subset of tasks, participants were given time to practice outside of the scanner to familiarize themselves with the new tasks and their instructions.

**Table 1.** Brief descriptions of the 22 tasks used in the present study, with an emphasis on the experimental (rather than control) task conditions.

fMRI task details					
Task	Description	Citation	Task order (sessions completed)	Run length (# of runs)	Total trials per block type^†^
Color-shape	Participants judge either the color or shape of a stimulus.	(Hakun et al., 2015)	1 (Sessions 1 & 2)	5:18 (1)	Single task 1: 14 Single task 2: 14 Switch: 35
Number-letter	Participants see a number-letter pair (e.g., “4M”) and choose whether the number is even in value, or whether the letter is a vowel, depending on the pair’s location on the screen.	(Reineberg et al., 2018)	2 (Sessions 1 & 2)	5:34 (1)	Single task 1: 21 Single task 2: 21 Switch: 35
Number-list	Participants shift between making judgments about the length or values in a number list (e.g., “444”).	(DiGirolamo et al., 2001)	3 (Sessions 1 & 2)	5:15 (1)	Single task 1: 21 Single task 2: 21 Switch: 42
Grid updating	Participants are shown a grid with a dot inside, followed by a series of instructions to mentally move around the grid. They then must choose whether an “X” in a final grid matches the position given by the instructions.	(Leung et al., 2007)	4 (Sessions 1 & 2)	5:59 (1)	Experiment: 10 Control: 10
Letter *N-*back	Participants view a series of letters and must select whether the current letter is the same as the one they saw two trials previously (2-back), or whether the current letter is “X” (0-back).	(Demetriou et al., 2018)	5 (Sessions 1 & 2)	5:45 (1)	2-Back: 60 0-Back: 60
Sternberg	Participants view a list of letters, followed by a single probe letter. They must indicate whether the probe letter was in the previous list.	(Altamura et al., 2007)	6 (Sessions 1 & 2)	5:50 (1)	Experiment: 24 Control: 20
Color-word Stroop	In the incongruent condition, participants respond to the ink color of an incongruent color word (e.g., BLUE in red ink).	(Kikuchi et al., 2012)	7 (Sessions 1 & 2)	5:21 (1)	Incongruent: 60 Neutral: 60
Simon	Participants are shown an arrow on either the left or right side of the screen, and they must respond to the direction in which the arrow faces.	(Georgiou-Karistianis et al., 2007)	8 (Sessions 1 & 2)	5:21 (1)	Experiment: 36 Control: 24
Color flanker	Participants view a row of colored circles and respond to the color of the central circle.	(Wager et al., 2005)	9 (Sessions 1 & 2)	5:49 (1)	Incongruent: 72 Congruent: 72
Inhibit, switch, update	Participants complete a series of tasks designed to measure inhibition, set shifting, and working memory updating via rule changes, where they make judgments about a row of colored squares.	(Lemire-Rodger et al., 2019)	1 (Sessions 3 & 4)	10:10 (4-5*)	Control: 133-166 Update: 134-168 Inhibit: 133-166 Switch: 32-40**
Counter switching	Participants keep counts of the number of left- and right-facing arrows presented. At the end of the trial, they are given a left or right cue and must indicate the number of arrows they saw.	(Sylvester et al., 2003)	1 (Sessions 5 & 6)	6:20 (1)	Experiment: 66 Control: 66
Stimulus-response compatibility	Participants see a centrally presented arrow facing either left or right. Their goal is to press the opposite hand’s button (e.g., when viewing a left-facing arrow, they use their right hand).	(Sylvester et al., 2003)	2 (Sessions 5 & 6)	5:56 (1)	Experiment: 66 Control: 66
Spatial working memory	Participants are shown a series of squares distributed across a blank screen. After a delay, they are shown a probe square and indicate whether it is in the same position as a square from the previously seen display.	(Silk et al., 2010)	3 (Sessions 5 & 6)	5:40 (1)	Experiment: 12 Control: 12
Overt attention	Participants are shown a rotating cross with a single red arm in the center of eight white circles. They must fixate on the central cross, while deciding if the red arm is pointing to a circle that has become smaller in size. If so, they make a saccade to the smaller circle.	(de Haan et al., 2008)	4 (Sessions 5 & 6)	6:20 (1)	Experiment: 120 Control: 120
Covert attention	Participants are shown a rotating cross with a single red arm in the center of eight white circles. They must fixate on the central cross, while deciding if the red arm is pointing to a circle that has become smaller in size. If so, they make a button press to indicate the change while remaining fixated on the central cross.	(de Haan et al., 2008)	5 (Sessions 5 & 6)	6:20 (1)	Experiment: 120 Control: 120
Random number generation	Participants are shown a flashing cue. In time with the cue, they press buttons in a random order.	(Gilbert et al., 2008)	6 (Sessions 5 & 6)	6:00 (1)	Experiment: 150 Control: 150
Go/No-Go	Participants are shown a series of letters and asked to respond with a single button to each, unless the letter is “X”. Then, they make no response.	(Wager et al., 2005)	7 (Sessions 5 & 6)	5:57 (1)	Experiment: 120 Control: 120
Item recognition	Participants view a set of 4 uppercase letters, which is replaced by a single, lowercase probe. They indicate whether the probe is one of the 4 previously seen letters.	(Awh et al., 1996)	8 (Sessions 5 & 6)	6:20 (1)	Experiment: 24 Control: 24
Number Stroop	Participants are shown a list of numbers (e.g., “33”) and asked to indicate the length of the list, rather than the value of the numbers.	(Shilling et al., 2002)	1 (Sessions 7 & 8)	6:00 (1)	Incongruent: 60 Neutral: 60
Arrow Stroop	Participants are shown an arrow with a non-matching direction word inside (e.g., a left-facing arrow with the word “RIGHT” inside). They must respond to the direction of the arrow, rather than the word.	(Shilling et al., 2002)	2 (Sessions 7 & 8)	6:00 (1)	Incongruent: 60 Neutral: 60
Combined Stroop	This task is a combination of the Number Stroop and Arrow Stroop tasks, in which blocks alternate between the two.	(Shilling et al., 2002)	3 (Sessions 7 & 8)	6:00 (2)	Arrow incongruent: 120 Number incongruent: 120
Executive function	Participants complete a series of tasks designed to measure inhibition, set shifting, and working memory updating via rule changes, in which they make judgments about a series of numbers and color words.	(Saylik et al., 2022)	4 (Sessions 7 & 8)	~13:00*** (2)	Control 1: 60 Control 2: 60 Control 3: 60 Update: 100 Inhibit: 100 Switch: 100

† Values indicate the total number of trials across all blocks of that type (across all runs). When block types have clear names (e.g., “Incongruent” and “Congruent”), these are used. Otherwise, “Experiment” and “Control” are used to distinguish the block types.

* One participant completed only 4 runs of the ISU task during session 3 due to illness.

** Switching trials were modeled as the first trial of each block (not including the first block of the run).

*** The Executive Function task was split into two runs (Run 1: 13:15, Run 2: 12:55) to accommodate the scanner.

All tasks were created using PsychoPy (Peirce et al., 2019). All tasks were adapted from previously published studies and were selected to broadly measure various cognitive control abilities.

#### 2.1.4 fMRI data preprocessing

Data were preprocessed using FreeSurfer (Fischl, 2012). For each scanning session, each participant’s cortical surface was estimated from their T1 anatomical image using *recon-all,* including intensity normalization to correct for residual inhomogeneities during scan acquisition. Data were resampled from native space to the *fsaverage7* cortical mesh (containing 163,842 vertices per hemisphere). The resulting white and pial matter boundaries were manually inspected and compared to the original volumes. Deviations from the anatomical image were manually corrected, and *recon-all* was run a second time. Then, the FreeSurfer Functional Analysis Stream (FS-FAST) was used to perform preprocessing and first-level (individual) analyses. Preprocessing included motion-correction using the first volume of the first run, slice-timing correction, and application of a 5 mm full-width-at-half-maximum smoothing kernel. In addition, preprocessing (including *recon-all*) included field map corrections and intensity normalization to reduce effects from gain-field inhomogeneity. Finally, a general linear model (GLM) was fit for each first-level analysis (i.e., for each task from each scanning session). The GLMs for each task were modeled the same way, using SPM’s canonical hemodynamic response function (using *spmhrf 0* during *mkanalysis-sess*). Every model included one regressor per task condition, as well as one baseline/intercept term and two polynomial drift terms (linear and quadratic) per task run, which were included as nuisance covariates. Finally, 112 statistical contrasts were computed from the 22 tasks (Supplementary Table S1).

### 2.2 Data preparation and analysis

All data preparation and analyses were completed using MATLAB (version R2022a) or R (version 4.4.1), including the base R, *psych,* and *tidyr* packages as well as the *qqplotr* and *ggplot2* packages for visualization (Almeida et al., 2018; R Core Team, 2024; Revelle, 2024; Wickham, 2016; Wickham et al., 2024).

#### 2.2.1 Matrix creation

Contrast effect size (CES) data obtained from the first-level fMRI analyses were input into matrices using MATLAB R2022a for further analysis. Because the MCD dataset includes two sessions of data, we first randomly assigned the contrast data from each task run to either Set1 or Set2 datasets (this assignment was random to avoid systematic differences between the datasets due to practice effects). Contrast data assigned to Set1 from the left hemisphere for a given subject were horizontally concatenated, creating a vertex-by-contrast (163,842 x 112) matrix. This was repeated for the right hemisphere, and then the hemispheres were vertically concatenated to create a whole-brain (327,684 x 112) Set1 matrix for each person. The Set2 matrix was created in an identical manner, resulting in four Person/Set matrices (Person 1/ Set1 [P1S1]; Person 1/ Set2 [P1S2], Person 2/ Set1 [P2S1], and Person 2/ Set2 [P2S2]).

We then repeated this procedure for four anatomical measures that are computed at each vertex by Freesurfer: cortical thickness, curvature, surface area, and sulcal depth. Because each participant had multiple scan sessions, we randomized by assigning data from odd-numbered scans to Set1 and even-numbered scans to Set2. This resulted in 4 more matrices per person and dataset (one per anatomical measure) of size 327,684 x 4.

#### 2.2.2 Analysis of within- and between-person similarity

If patterns of neural activity during cognitive control tasks are person-specific and reliable, then the similarity of the functional contrast data should be significantly higher within the same individual than between different people, even after controlling for anatomical similarity. We tested this prediction in two ways. First, we analyzed similarity at the individual vertex level, assessing how similar each vertex behaved within-person and between-person across the 112 contrasts. Next, we analyzed the similarity of the whole brain contrast maps within-person and between-person.

##### 2.2.2.1 Vertex-based analysis

We first calculated similarity at each individual vertex separately for the functional contrast data and the anatomical measures. Some rows in Freesurfer’s output for the different measures include missing data. In our case, missing data was primarily located on the medial surface, near subcortical structures. These regions, while included on the *fsaverage7* surface, do not typically have data for cortical measures. These rows were removed from analysis to avoid missing cases in later steps, and so the vertex-based analysis included 299,522 vertices, rather than the full 327,684.

To calculate within-person similarity at each vertex, we calculated the zero-order Pearson correlation between corresponding *rows* of data in Set1 and Set2 in Person 1 (P1S1 vs. P1S2). For the contrast (functional) data, this entailed calculating the correlation across all 112 contrast values at each individual vertex. For the anatomical measures, this entailed calculating the correlation across the four scan sessions at each vertex. This procedure was repeated for Person 2 (P2S1 vs. P2S2), resulting in two correlation values (1 per person) at each vertex, which were then averaged to create a single mean similarity score per vertex. After calculating mean similarity scores for each of the five measures (functional data and four anatomical measures), the final output consisted of five separate vectors of within-person similarity scores, of size 299,522 x 1.

Between-person similarity was also defined as the average correlation between corresponding vertices, but this time between people. Specifically, for each vertex, we correlated the values at each vertex in Set1 for Person 1 with the corresponding values in Set1 for Person 2 (P1S1 vs. P2S1). We again repeated this procedure, this time computing the correlation between each vertex’s data in Set2 in Person 1 and the corresponding data in Set2 in Person 2 (P1S2 vs. P2S2). For each of the five measures, we then calculated a mean between-person similarity score at each vertex by averaging the correlations from Set1 and Set2. This similarly resulted in 5 separate vectors of between-person similarity scores of size 299,522 x 1. We then concatenated the within-person and between-person data to perform the regression analysis in the following section.

###### 2.2.2.1.1 Statistical analysis

We then regressed the functional similarity scores onto the four anatomical similarity scores (cortical thickness, surface area, curvature, and sulcal depth) and analyzed the residuals in order to control for anatomical similarity:

$$\begin{array}{l} Y=\beta_{0}+\;\beta_{1}\left(cortical\;thickness\right)+\;\beta_{2}\left(surface\;area\right)\\ \;\;\;\;\;\;\;\;\;+\;\beta_{3}\left(curvature\right)+\;\beta_{4}\left(sulcal\;depth\right)+\;\varepsilon \end{array}$$

Where Y represents the functional (contrast) similarity scores and epsilon represents the residuals. We confirmed that the assumptions of regression were met, including linearity, homoscedasticity, and normality of the residuals. We then conducted a permutation test with 500,000 iterations on the standardized residuals of the similarity scores (within- and between-person) to test whether within-person similarity would still be higher than between-person similarity, even after controlling for anatomical similarity. For each iteration, the labels were randomly shuffled and the mean difference was recalculated. The permutation *p*-value was defined as the proportion of iterations in which the permuted mean difference was as extreme or more extreme than the observed mean difference.

##### 2.2.2.2 Contrast-based analysis

We also analyzed similarity at the level of contrasts across the whole brain. In this case, we were not able to control for anatomical similarity, because we did not have separate anatomical measurements for every contrast. We first calculated the zero-order Pearson correlation between each contrast map (i.e., taking the correlation across *columns*) in Set1 and Set2 in Person 1 (P1S1 vs. P1S2). This procedure was repeated for Person 2 (P2S1 vs. P2S2). We then calculated the average within-person similarity for each contrast by taking the mean of Person 1 and Person 2’s correlations, resulting in 112 within-person similarity scores.

Between-person similarity was similarly computed as the average correlation between corresponding contrast maps in the two different individuals. We computed the correlation between each contrast map in Set1 in Person 1 with the corresponding contrast map in Set1 in Person 2 (P1S1 vs. P2S1) and repeated this procedure for Set2 (P1S2 vs. P2S2). We then averaged the two correlations for each contrast, resulting in 112 between-person similarity scores.

###### 2.2.2.2.1 Statistical analysis

We then performed a paired samples *t-*test on the within- and between-person similarity scores. And finally, in order to ensure that our two participants were not more different from each other than would be expected in a larger sample, we also calculated between-person similarity scores from the larger, publicly available Dual Mechanisms of Cognitive Control (DMCC55B) dataset (Etzel et al., 2022). We performed a Welch’s *t*-test (due to the unequal sample sizes) to test whether the between-person similarity in the present study was significantly greater than that found in the larger sample.

#### 2.2.3 Analysis of similarity across tasks

Finally, we tested whether within-person and between-person similarity scores differ based on task. To do so, we first assigned the 112 contrast-based within-person similarity scores (1 per contrast) to one of 22 groups, corresponding to the task from which the contrast was created. We then ran a one-way ANOVA to test whether within-person similarity varied by this task grouping variable. This procedure was then repeated for between-person similarity. Because an examination of the boxplots for both sets of similarity scores (see Figs. 3-4) suggested that the assumption of homogeneity of variance was violated, we used a Welch’s ANOVA. We then investigated the boxplots to examine which tasks might be best suited for individual differences (i.e., tasks with high within-person similarity and low between-person similarity). For each task, we calculated the within/between similarity difference by subtracting the median between-person similarity score from the corresponding median within-person similarity score to explore which tasks provided the best consistency within individuals while still differentiating between them. Finally, given that many tasks produce both high within-person similarity and high between-person similarity, we calculated the correlation between the two median vectors (between-person similarity by task and within-person similarity by task).

## 3 Results

### 3.1 Activation maps are significantly more similar within-person than between-person, even after controlling for anatomical similarity

The permutation test of the standardized residuals demonstrated that, after 500,000 iterations, within-person contrast similarity (M = 0.43, SD = 0.82) was found to be greater than between-person similarity (M = -0.43, SD = 0.98), *p* < 0.001, even after controlling for anatomical similarity (Supplementary Fig. S1). The range of the permuted differences was between -0.01 and 0.01. The effect size was large (Cohen’s d = 0.94), indicating a pronounced difference in means that is very unlikely to have occurred by chance.

**Fig. 1.**
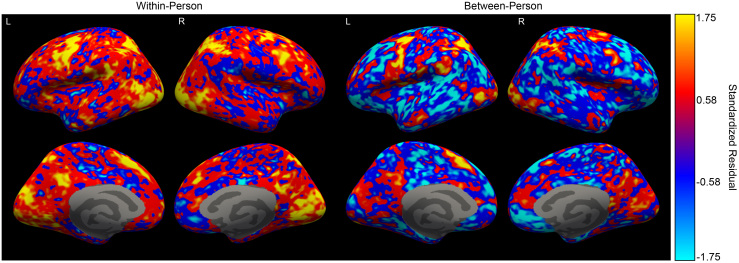
Visualization of Regression Residuals. Brain maps of the standardized residuals derived from the regression model predicting functional (contrast) similarity from anatomical similarity. Within-person similarity (on the left) is greater than what would be predicted by anatomical similarity across most of the cortical surface. Conversely, between-person similarity (on the right) is less than what would be predicted by anatomical similarity across most of the cortical surface. Red and yellow values indicate positive (and high positive) values, whereas blue colors indicate negative values (with light blue representing the lowest negative values). The color scale is consistent across within- and between-person panels. The top row displays the lateral surface, while the bottom row displays the medial surface. Gray medial regions are not included in the cortical surface, and therefore were not included in the analysis.

We further created brain maps of the within- and between-person residuals on the *fsaverage7* cortical surface to further highlight this difference (Fig. 1; see Supplementary Fig. S2 for brain maps of similarity scores prior to regression). Red and yellow hues in this figure represent higher numbers, indicating regions where contrast similarity is higher than would be predicted by anatomical similarity alone. In contrast, bright blue regions in these maps indicate regions where contrast similarity is lower than would be predicted by anatomical similarity. As the figure illustrates, within-person contrast similarity is higher than what anatomical similarity would predict throughout most of the cortical surface. In contrast, between-person similarity is lower than what anatomical similarity would predict throughout most of the cortex.

The contrast-based similarity analysis produced a similar pattern of results. The *t*-test indicated that within-person similarity scores were significantly larger (M = 0.37, SD = 0.2) than between-person similarity scores (M = 0.18, SD = 0.13), *t*(111) = 14.68, *p* < 0.001. The effect size of this difference was also quite large (Cohen’s d = 1.11). In addition, between-person similarity calculated from the 55 people in the DMCC55B dataset (M = 0.20, SD = 0.15) was quite similar to the between-person similarity in our dataset (M = 0.18, SD = 0.13), and the difference between these two measures was not significant (*t*(37.68) = 0.56, *p* = 0.58). In short, the contrast similarity between the two participants in the present study is comparable to the contrast similarity between pairs of participants in a larger sample.

**Fig. 2.**
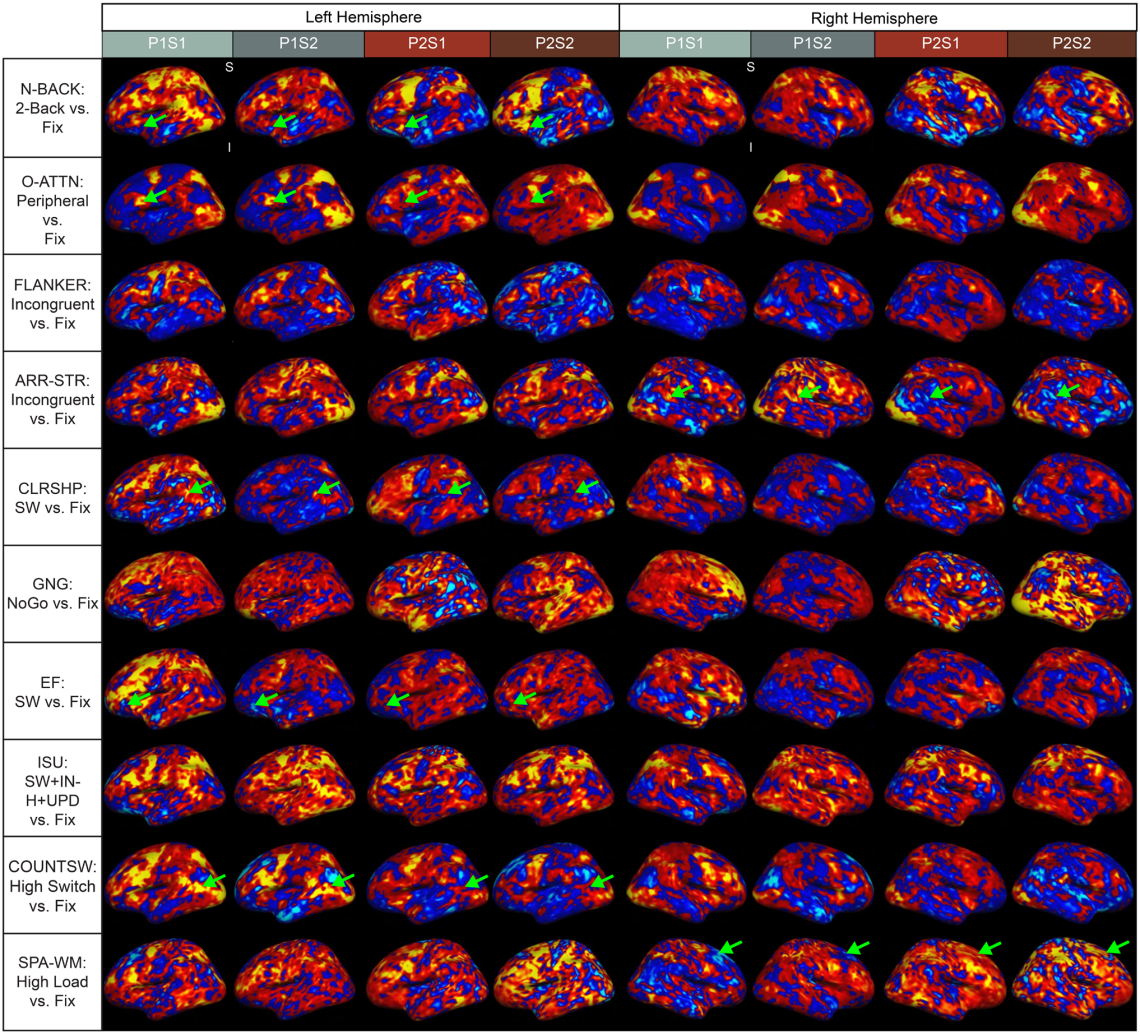
Contrasts Are More Similar Within an Individual*.* A selection of 10 (of 112) first-level (individual) contrast maps from the two individuals, in order to highlight qualitative similarities and differences in patterns of neural activity. The color scale varies across images to optimize individual contrast visualization, with a maximum intensity corresponding to a –log10(*p*) value of 5 (*p* = 10^—5^); therefore, color intensity indicates relative activation and does not correspond to identical numeric values across maps. Warm colors indicate positive values, or more activation during the experimental condition, whereas cool tones indicate negative values, or more activation during the baseline condition. Green arrows highlight differences between the two individuals. N-BACK = Letter N-Back, O-ATTN = Overt Attention, ARR-STR = Arrow Stroop, CLRSHP = Color-Shape, SW = switching (also called shifting or set shifting), GNG = Go/No-Go, EF = Executive Function, ISU = Inhibit, Switch, Update, INH = inhibition, UPD = updating, COUNTSW = Counter Switching, SPA-WM = Spatial Working Memory.

The difference between within-person and between-person similarity is also apparent upon visual inspection of brain activation patterns. Figure 2 displays 10 randomly selected contrasts (from the 112 total contrasts) across the four datasets (P1S1, P1S2, P2S1, and P2S2). In most contrasts (but not all; see section 3.2), the contrast maps appear more similar when they are from the same person. In Figure 2, green arrows highlight a selection of regions in which contrast activity appears quite different in the two people despite being consistent within each person. In fact, there are some instances in which the two individuals displayed opposite patterns. For example, when contrasting the experimental (“high switch”) condition against the fixation condition in the Counter-Switching task, Person 1 displayed increased activity near the middle left temporal lobe; in this same region, Person 2 exhibited deactivation (Fig. 2; row 9, green arrows). Similarly, during a contrast from the Spatial Working Memory task that contrasted the high load condition from the task with fixation, Person 2 displayed increased activation in the right superior frontal lobe, but Person 1 did not (Fig. 2; row 10, green arrows).

### 3.2 Similarity of contrast data changes with task

**Fig. 3.**
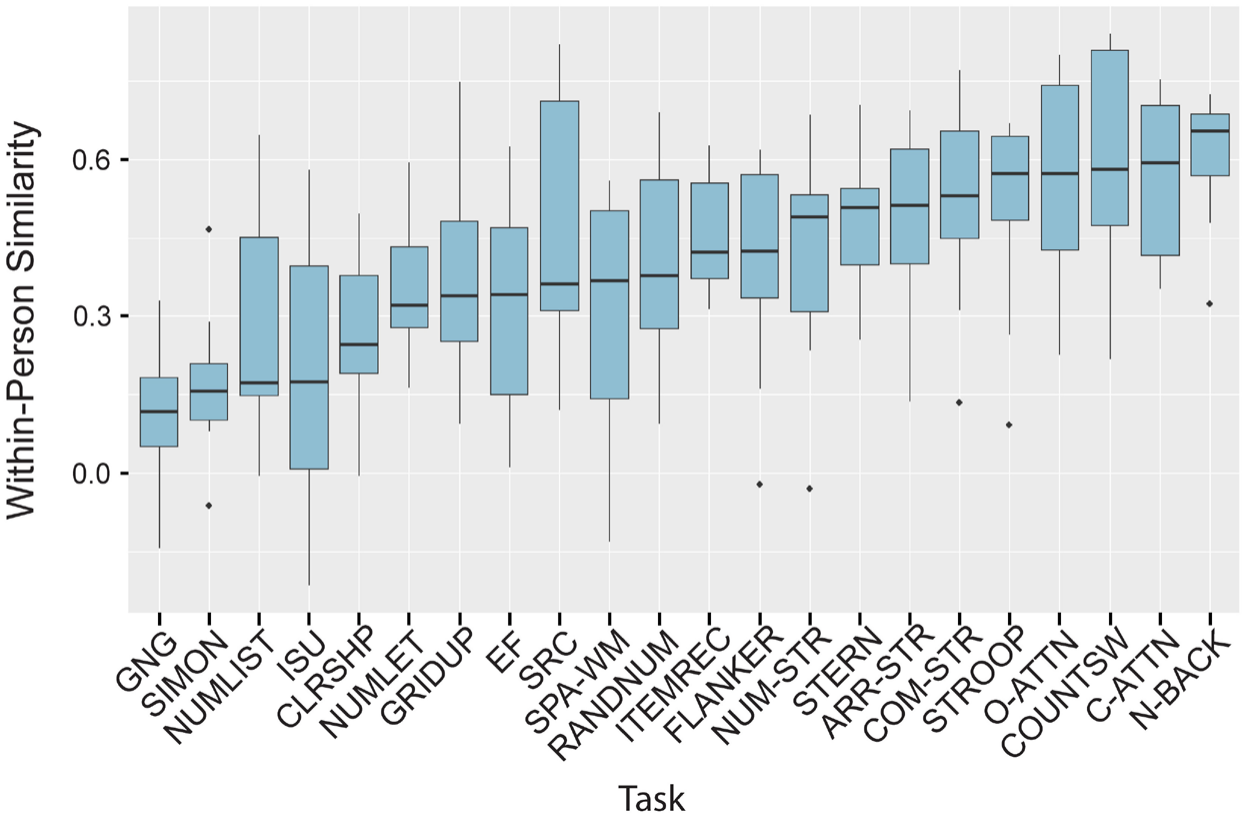
Within-Person Similarity by Task*.* Boxplot displaying within-person similarity scores across the 22 cognitive control tasks, sorted from lowest (left) to highest (right). The box midline indicates the median, while box edges define the interquartile range. Solid lines reflect 1.5x the interquartile range, and values beyond those are indicated with solid dots. GNG = Go/No-Go, NUMLIST = Number-List, ISU = Inhibit, Switch, Update, CLRSHP = Color-Shape, NUMLET = Number-Letter, GRIDUP = Grid Updating, EF = Executive Function, SRC = Stimulus-Response Compatibility, SPA-WM = Spatial Working Memory, RANDNUM = Random Number Generation, ITEMREC = Item Recognition, NUM-STR = Number Stroop, STERN = Sternberg, ARR-STR = Arrow Stroop, COM-STR = Combined Stroop, O-ATTN = Overt Attention, COUNTSW = Counter Switching, C-ATTN = Covert Attention, N-BACK = Letter *N*-back.

The one-way ANOVA comparing the effects of task on within-person similarity revealed significant differences among the tasks (*F*(21, 63.43) = 5.20, *p* < 0.001), suggesting that some tasks produce more consistent, or more reliable, patterns of activity in a given person compared with others. For example, the Letter *N*-Back task has the highest average within-person similarity of all the tasks; patterns of neural activity from this task are highly correlated when they come from the same individual (Fig. 3; see Supplementary Figs. S3-S4 for individual within-person data).

**Fig. 4.**
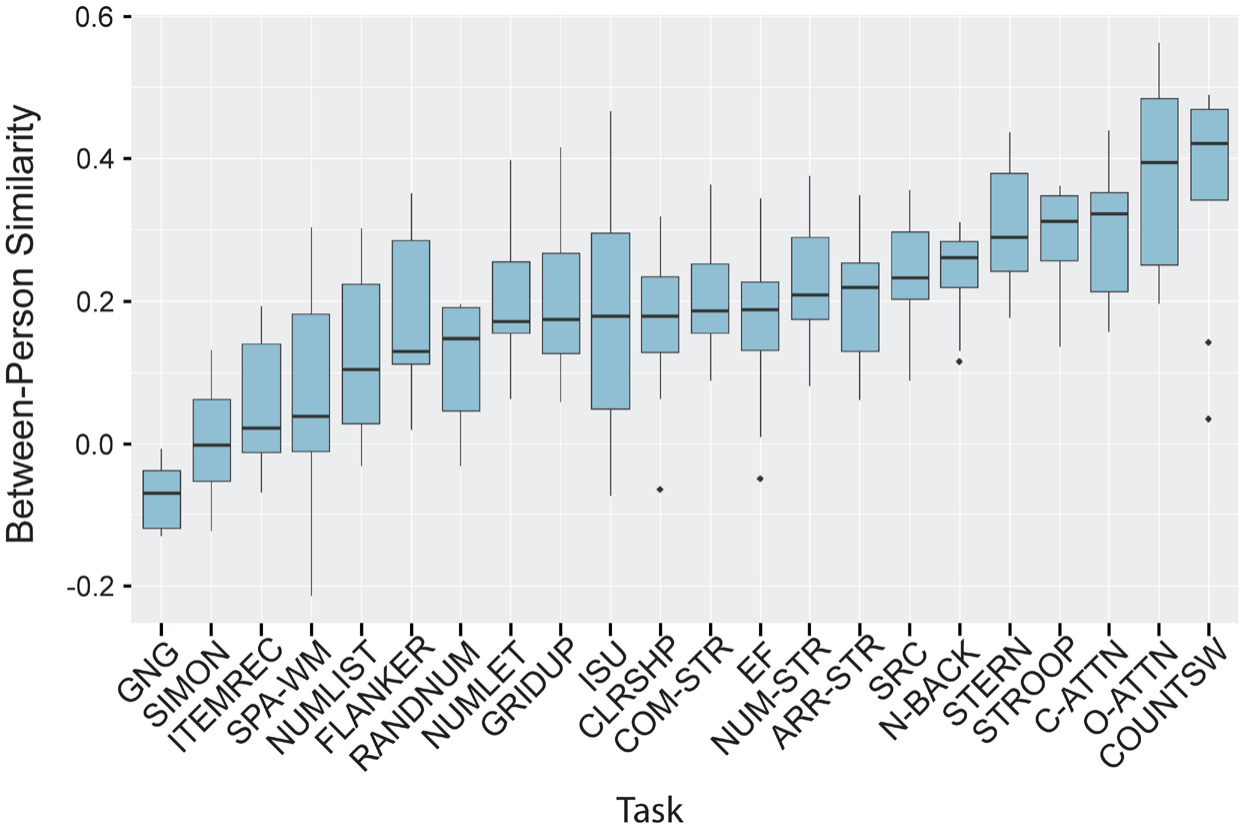
Between-Person Similarity by Task. Boxplot displaying between-person similarity scores across the 22 cognitive control tasks, sorted from lowest (left) to highest (right). The box midline indicates the median, while box edges define the interquartile range. Solid lines reflect 1.5x the interquartile range, and values beyond those are indicated with solid dots. GNG = Go/No-Go, ITEMREC = Item Recognition, SPA-WM = Spatial Working Memory, NUMLIST = Number-List, RANDNUM = Random Number Generation, NUMLET = Number-Letter, GRIDUP = Grid Updating, ISU = Inhibit, Switch, Update, CLRSHP = Color-Shape, COM-STR = Combined Stroop, EF = Executive Function, NUM-STR = Number Stroop, ARR-STR = Arrow Stroop, SRC = Stimulus-Response Compatibility, N-BACK = Letter *N*-Back, STERN = Sternberg, C-ATTN = Covert Attention, O-ATTN = Overt Attention, COUNTSW = Counter Switching.

A similar pattern was found for between-person similarity, (*F*(21, 63.79) = 13.10, *p* < 0.001), suggesting that some tasks make individuals’ brains look more similar to each other. For example, the Counter-Switching, Overt and Covert Attention, and Stroop tasks have the highest between-person similarity of all the tasks, suggesting that the patterns of neural activity from the two individuals are more similar during these tasks. On the other hand, the Go/No-Go, Simon, Item Recognition, and Spatial Working Memory tasks have the lowest between-person similarity (Fig. 4; see Supplementary Figs. S5-S6 for individual set1/set2 data).

Importantly, within-person similarity tends to be higher across all tasks, but some tasks have relatively high within-person similarity and low between-person similarity (Figs. 3-4; Table 2). In fact, after subtracting median between-person similarity scores from within-person similarity scores on each task, five tasks stood out as having the highest within-between difference: the Flanker (within-between difference = 0.3), Spatial Working Memory (0.33), Combined Stroop (0.34), Letter *N*-Back (0.39), and Item Recognition (0.4) tasks (Table 2). Higher scores reflect greater within-person similarity compared to between-person similarity, and therefore suggest that these tasks consistently produce the same results within a given person, but also consistently differentiate individuals. These tasks might be better suited for individual differences research compared to the other 17 in our task battery.

**Table 2.** Table displaying the median within- and between-person similarity scores for each task.

Within – between similarity by task			
Task	Within-person similarity (med.)	Between-person similarity (med.)	Within/between difference
Inhibit, switch, update (ISU)	0.17	0.18	-0.01
Color-shape (CLRSHP)	0.25	0.18	0.07
Number-list (NUMLIST)	0.17	0.1	0.07
Stimulus-response Compatibility (SRC)	0.36	0.23	0.13
Number-letter (NUMLET)	0.32	0.17	0.15
Executive function (EF)	0.34	0.19	0.15
Simon (SIMON)	0.16	0	0.16
Counter switching (COUNTSW)	0.58	0.42	0.16
Grid updating (GRIDUP)	0.34	0.17	0.17
Overt attention (O-ATTN)	0.57	0.39	0.18
Go/No-Go (GNG)	0.12	-0.07	0.19
Sternberg (STERN)	0.51	0.29	0.22
Random number generation (RANDNUM)	0.38	0.15	0.23
Color-word Stroop (STROOP)	0.57	0.31	0.26
Covert attention (C-ATTN)	0.59	0.32	0.27
Number Stroop (NUM-STR)	0.49	0.21	0.28
Arrow Stroop (ARR-STR)	0.51	0.22	0.29
Color Flanker (FLANKER)	0.43	0.13	0.3
Spatial working memory (SPA-WM)	0.37	0.04	0.33
Combined Stroop (COM-STR)	0.53	0.19	0.34
Letter *N-*back (N-BACK)	0.65	0.26	0.39
Item recognition (ITEMREC)	0.42	0.02	0.4

Column 4 displays the within/between difference (within-person median minus between-person median). Some tasks have higher within-person similarity and lower between-person similarity, which is preferred for individual differences research.

Other tasks produced low between-person similarity, and also low within-person similarity. For example, the Go/No-Go and Simon tasks had the lowest similarity scores in both measures. Although they might differentiate between people well, they also lack reliability within a given individual and may be unreliable tasks overall. We also observed a strong correlation between the median between- and within-person similarity scores (r = 0.74), suggesting that many tasks that produce reliable activations within individuals also look quite similar across people. While these tasks may be useful for typical experimental research, they may not be ideal for individual differences research (Hedge et al., 2018).

## 4 Discussion

In the present study, we leveraged a novel approach combining precision neuroimaging with a large task battery to examine interindividual differences in control-related patterns of neural activity. We repeated this analysis in a larger sample as well, finding that our two densely-imaged participants are not more different from each other than would be expected. Finally, we also compared these similarity measures as a function of task to explore how the similarity of activation patterns changes across different tasks. We found that cognitive control-related patterns of neural activity are significantly more similar when they come from the same person than from another person, even after controlling for differences in brain anatomy. We also found that the similarity of these patterns, whether from the same person or from different people, differs with changes in task, highlighting how different tasks might be better suited to individual-differences research than others.

Our findings build on previous person-specific and precision neuroimaging work by demonstrating that person-specific neural features are evident not only in connectivity profiles and functional parcellations, as prior studies have shown, but also in contrast maps, which reflect direct task-evoked neural activity (Braga & Buckner, 2017; Braga et al., 2020; DiNicola et al., 2020; Gordon et al., 2017; Gordon & Nelson, 2021; C. Gratton, Laumann, et al., 2018; Laumann et al., 2015; Seitzman et al., 2019). While previous work has established that person-specific features exist in multiple forms and are likely present in all individuals, to our knowledge, no study has yet extensively examined spatial variability so densely in a single cognitive domain. However, some such studies have suggested that interindividual differences in brain organization might be related to individual differences in behavior (Seitzman et al., 2019), something we were unable to explore in the present study. Future work might seek to investigate such questions, especially regarding cognitive control.

Our finding that both within-person and between-person similarity measures vary by task is also consistent with prior work suggesting that tasks may provide different information about the brain’s neural organization than resting state fMRI (rs-fMRI) alone (Finn, 2021; Finn et al., 2017). The bulk of precision neuroimaging work thus far has relied on rs-fMRI, in part because rs-fMRI can often reproduce results obtained with task-based fMRI as well as predict those results (Cole et al., 2014; Osher et al., 2019; Tavor et al., 2016; Tobyne et al., 2018). However, the finding that different tasks make patterns of activation look more or less similar across individuals highlights two important points regarding task-based fMRI.

The first concerns reliability, and what reliability means in different types of research. In some studies, reliability means that the same effect can be consistently reproduced in different experiments at different sites with different participants. For example, the present study included the color-word Stroop task. The Stroop effect, or the slowing of responses when participants view a color word in an incongruent ink color, is very reliable in the sense that it has been replicated many times (Hedge et al., 2018). This form of reliability involves high between-person similarity, which we also see in brain responses to the Stroop task (note the high between-person similarity of the Stroop task in Fig. 4). However, high between-person similarity has limited utility for individual-differences research. When studying individuals, the goal is often to obtain a reliable effect within a person while consistently differentiating individuals (i.e., high within-person similarity and low between-person similarity; (Hedge et al., 2018)). In the present study, we found that some cognitive control tasks might be better suited for this purpose than others; for example, the Item Recognition task was found to have the highest within/between similarity difference (Awh et al., 1996). This finding suggests that this task does a good job of differentiating between individuals while producing consistent effects within individuals. Future research might further explore what makes a cognitive task useful when studying individual differences, and might even consider devoting special attention to novel tasks designed for individual differences research, alongside changes in measurement and statistical approaches (Hedge et al., 2018).

Second, the finding that the similarity of neural activity patterns differs across tasks highlights the necessity of studying the brain under many conditions to obtain a truly detailed characterization of brain function and organization. Thirion et al. (2021) argue that the function of a given brain region cannot be known with certainty until that region’s response to a wide variety of contexts has been measured. Given the lack of consensus about the neural organization of cognitive control, using large task batteries to densely image cognitive control and identify the functions of brain regions associated with it may provide a more comprehensive understanding of how cognitive control is implemented in the brain (Badre & Nee, 2018; Smith et al., 2021). Therefore, future research might aim to take a similar approach to that of the present study to explore the neural organization of cognitive control.

One important limitation of the current study is that it only involved two participants, which obviously limits our ability to generalize the results to other people. On the other hand, limiting the number of participants allowed us to collect much more data within each person (112 contrasts from 22 tasks collected twice in each person). The participants, in this case, are more like the conditions in a traditional experiment, and the large number of contrasts within each person are more like the sample (and from that point of view, the sample is fairly large). While this design obviously does not allow us to relate individual differences in neural activity to individual differences in behavior, it does still provide a detailed description of how patterns of neural activity differ across different tasks. In addition, any potential lack of generalizability in our results is offset by our comparison of the present dataset to the DMCC55B dataset, in which we demonstrated that the between-person similarity of our two individuals is not significantly different than that which is found in larger samples. The DMCC55B dataset does not include as large of a task battery, nor does it include separate sessions from the same individuals, and so we were not able to repeat our other analyses in this larger sample. However, the fact that between-person similarity did not significantly differ across the two suggests that our other results might be replicable in larger samples as well. And finally, we might argue that our approach, wherein the focus is on dense sampling of individual participants, is not a limitation at all. Most previous neuroimaging research has relied on averaging across large numbers of individuals, but precision neuroimaging work, as well as work in mathematics, has demonstrated that averaging across individuals can provide a misleading representation of individual brains, and might contribute to our limited understanding of cognitive control (Braga & Buckner, 2017; DiNicola et al., 2020; Gordon et al., 2017; Gordon & Nelson, 2021; Michon et al., 2022; Molenaar, 2004; Molenaar & Campbell, 2009; Newbold et al., 2020; Seghier & Price, 2018; Seitzman et al., 2019; Sidman, 1952; Smith et al., 2021). Understanding cognitive control at the level of the individual instead could provide new insights that are not able to be studied using more traditional approaches.

Another limitation involves the tradeoff between collecting multiple runs of a single fMRI task, or a single run of many tasks, as was done in the present study. One important consideration with fMRI is collecting enough data to obtain reliable measurements of brain activity. This is one of the great advantages of precision neuroimaging; the repeated scanning in individuals allows for highly reliable measurements (Gordon et al., 2017; Laumann et al., 2015, 2017; Michon et al., 2022). However, most previous precision neuroimaging studies have collected hours of rs-fMRI data, or a small number of tasks. We opted to take an alternative approach, in which we instead collected 12 hours of data from individuals as they performed 22 different tasks, completing each of those tasks twice in different sessions. Collecting more data from each task may lead to even more reliable results, but doing so would be challenging with such a large number of tasks administered twice. In any case, we still observed significant differences between within-person and between-person similarity even though the data collected within each task was limited.

Finally, in the current study, we were unable to assess whether the differences we see in the two individuals (Fig. 2) are due to differences in cognitive processing or spatial organization. In other words, it is difficult to know whether participants used different cognitive processes during the tasks, or whether they used the same cognitive process, but those processes are organized differently across their brains. While we tried to account for potential differences in strategy (i.e., using a different cognitive process) by allowing the participants time to practice and using very specific task instructions, these two possibilities are difficult to distinguish in any study of interindividual differences. Given that recent precision neuroimaging work has found interindividual variability in cortical organization (e.g., Gordon et al., 2017; Gordon & Nelson, 2021), future work might seek to disentangle these possibilities by exploring the structure of cognitive processing in individuals.

In conclusion, we present a novel dense neuroimaging method that combines precision neuroimaging with an unusually large task battery in order to expand upon previous work focused on resting-state fMRI. The finding that the similarity of activation patterns is significantly higher within-person than between-person, even after controlling for anatomical similarity, is consistent with the hypothesis that control-related brain activity is both person-specific and reliable. We also demonstrated that both between- and within-person similarity in patterns of neural activity vary across different tasks, suggesting that some tasks might be better suited to individual differences research than others. This finding has important implications for cognitive task design, as only a few tasks seem to provide between-person differences while maintaining high within-person reliability, which is optimal for individual differences research. Together, these results highlight the value of using this novel neuroimaging approach to better understand the brain’s neural organization, including the potential of using a wide variety of task states to obtain a detailed characterization of brain function. Such approaches may be instrumental in furthering our understanding of, and addressing gaps in knowledge surrounding, the neural organization of cognitive control.

## Supplementary Material

Supplementary Material

## Data Availability

The data and code used in this study are not currently publicly available. Interested researchers may contact the authors for further information.
