# Supplementary material for: Brain activation during cognitive control tasks differs substantially between people but is reliable within individuals
Source: Imaging Neurosci (Camb). 2025 Nov 10;3:IMAG.a.995. doi: 10.1162/IMAG.a.995 (PMC12603659; doi:10.1162/IMAG.a.995)
Supplement: Supplementary Material [file IMAG.a.995_supp.pdf]

Supplementary Information

Table 1

List of fMRI Contrasts

Task (Citation)	Contrasts Computed
**Color-Shape**	Color vs. Fixation
(Hakun et al., 2015)	Shape vs. Fixation
	Color+Shape vs. Fixation
	Shifting vs. Fixation
	Shifting vs. Shape
	Shifting vs. Color
	Shifting vs. Color+Shape
**Number-Letter**	Letter vs. Fixation
(Reineberg et al., 2018)	Number vs. Fixation
	Number+Letter vs. Fixation
	Shifting vs. Fixation
	Shifting vs. Letter
	Shifting vs. Number
	Shifting vs. Number+Letter
**Number-List**	Length vs. Fixation
(DiGirolamo et al., 2001)	Value vs. Fixation
	Value+Length vs. Fixation
	Shifting vs. Fixation
	Shifting vs. Value
	Shifting vs. Length
	Shifting vs. Value+Length
**Grid Updating**	Fixation vs. TASK
(Leung et al., 2007)	CTRL vs. Fixation
	Updating vs. Fixation
	Updating vs. CTRL
**Letter *N*-back**	Fixation vs. TASK
(Demetriou et al., 2018)	2-Back vs. Fixation
	0-Back vs. Fixation
	2-Back vs. 0-Back
**Sternberg**	Fixation vs. TASK
(Altamura et al., 2007)	CTRL vs. Fixation
	EXP vs. Fixation
	EXP vs. CTRL
**Color-Word Stroop**	Fixation vs. TASK
(Kikuchi et al., 2012)	Incongruent vs. Fixation
	Congruent vs. Fixation
	Incongruent vs. Congruent
**Simon**	Fixation vs. TASK
(Georgiou-Karistianis et al., 2007)	CTRL vs. Fixation
	EXP vs. Fixation
	EXP vs. CTRL
**Color Flanker**	Fixation vs. TASK
(Wager et al., 2005)	Incongruent vs. Fixation
	Congruent vs. Fixation

	Incongruent vs. Congruent
**Inhibit, Switch, Update**	INH vs. Fixation
(Lemire-Rodger et al., 2019)	SW vs. Fixation
	UPD vs. Fixation
	INH vs. CTRL
	SW vs. CTRL
	UPD vs. CTRL
	INH vs. SW+UPD
	SW vs. INH+UPD
	UPD vs. SW+INH
	INH+UPD+SW vs. Fixation
	INH+UPD+SW vs. CTRL
**Counter Switching**	TASK vs. Fixation
(Sylvester et al., 2003)	High Switch vs. Fixation
	Low Switch vs. Fixation
	High Switch vs. Low Switch
**Stimulus-Response Compatibility**	TASK vs. Fixation
(Sylvester et al., 2003)	Opposite vs. Fixation
	Same vs. Fixation
	Opposite vs. Same
**Spatial Working Memory**	TASK vs. Fixation
(Silk et al., 2010)	High Load vs. Low Load
	High Load vs. Fixation
	Low Load vs. Fixation
**Overt Attention**	TASK vs. Fixation
(de Haan et al., 2008)	CTRL vs. Fixation
	EXP vs. CTRL
	EXP vs. Fixation
**Covert Attention**	TASK vs. Fixation
(de Haan et al., 2008)	CTRL vs. Fixation
	EXP vs. CTRL
	EXP vs. Fixation
**Random Number Generation** (Gilbert et al., 2008)	TASK vs. Fixation
	CTRL vs. Fixation
	EXP vs. Fixation
	EXP vs. CTRL
**Go/No-Go**	TASK vs. Fixation
(Wager et al., 2005)	Go vs. Fixation
	NoGo vs. Fixation
	NoGo vs. Go
**Item Recognition**	TASK vs. Fixation
(Awh et al., 1996)	CTRL vs. Fixation
	EXP vs. Fixation
	EXP vs. CTRL
**Number Stroop**	TASK vs. Fixation
(Shilling et al., 2002)	Neutral vs. Fixation
	Incongruent vs. Fixation
	Incongruent vs. Neutral
**Arrow Stroop**	TASK vs. Fixation
(Shilling et al., 2002)	Neutral vs. Fixation
	Incongruent vs. Fixation
	Incongruent vs. Neutral
**Combined Stroop**	TASK vs. Fixation

(Shilling et al., 2002)	Incongruent (ASTR) vs. Incongruent (NSTR)
	Incongruent (ASTR) vs. Fixation
	Incongruent (NSTR) vs. Fixation
**Executive Function**	CTRL1 vs. Fixation
(Saylik et al., 2022)	CTRL2 vs. Fixation
	CTRL3 vs. Fixation
	INH vs. Fixation
	SW vs. Fixation
	UPD vs. Fixation
	INH vs. CTRL3
	SW vs. CTRL1+CTRL2
	UPD vs. CTRL3
	INH vs. SW
	UPD vs. INH
	UPD vs. SW

Table S1. List of the 112 contrasts that were computed for each task (see **2.1.4 fMRI Data Preprocessing**).

Figure S1

Permutation Test on Standardized Residuals

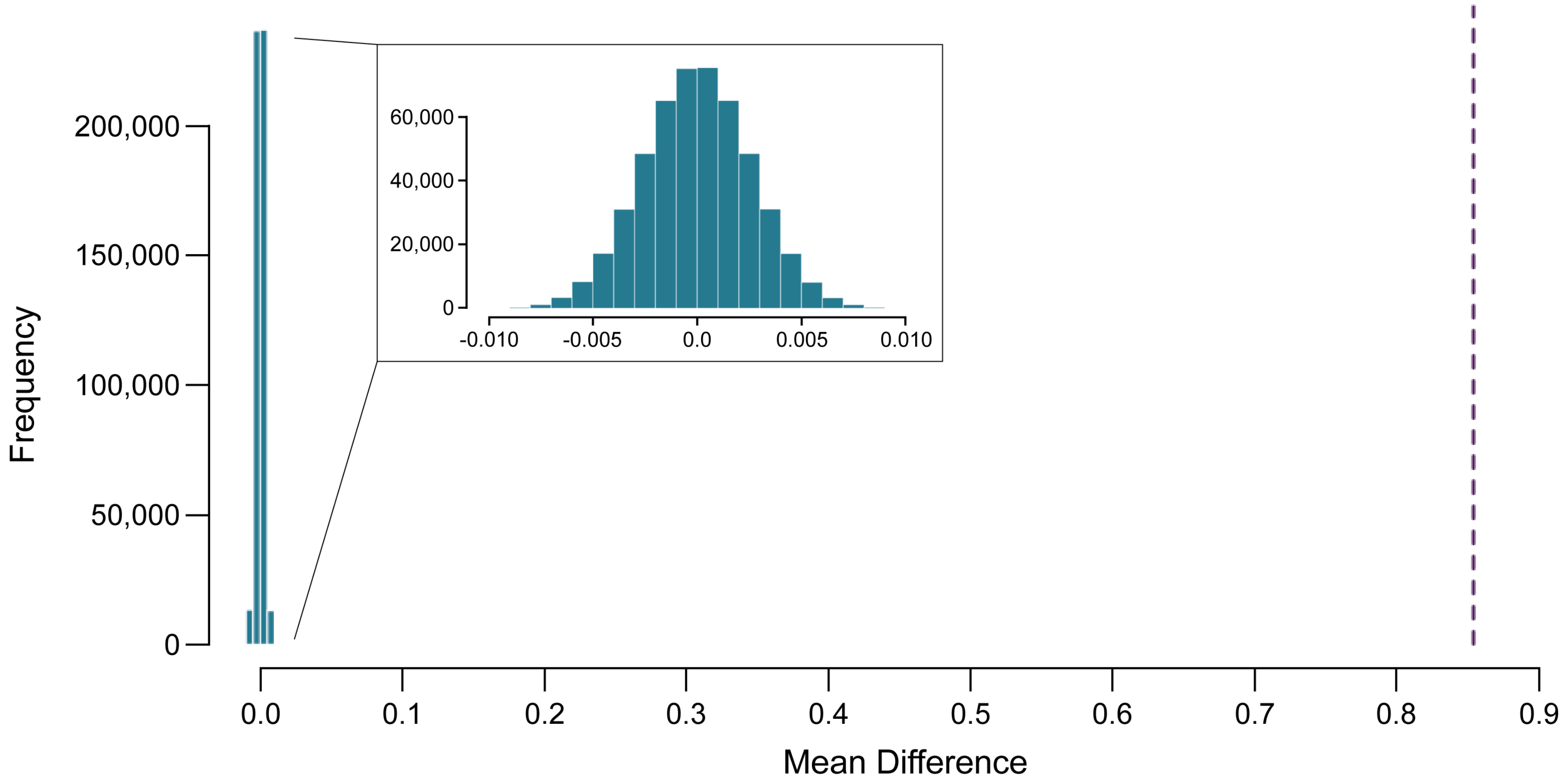

Figure S1: Histogram displaying results of the permutation test conducted on the standardized residuals from the regression model predicting functional similarity from anatomical similarity. The blue histogram bars represent the null distribution of permuted mean differences over 500,000 iterations. The purple dashed line represents the observed mean difference between within- and between-person standardized residuals. As shown by the histogram, none of the 500,000 iterations produced a mean difference as extreme as our observed mean difference, indicating that our results are highly unlikely to have occurred by chance.

Figure S2

Visualization of Similarity Scores Before Regression

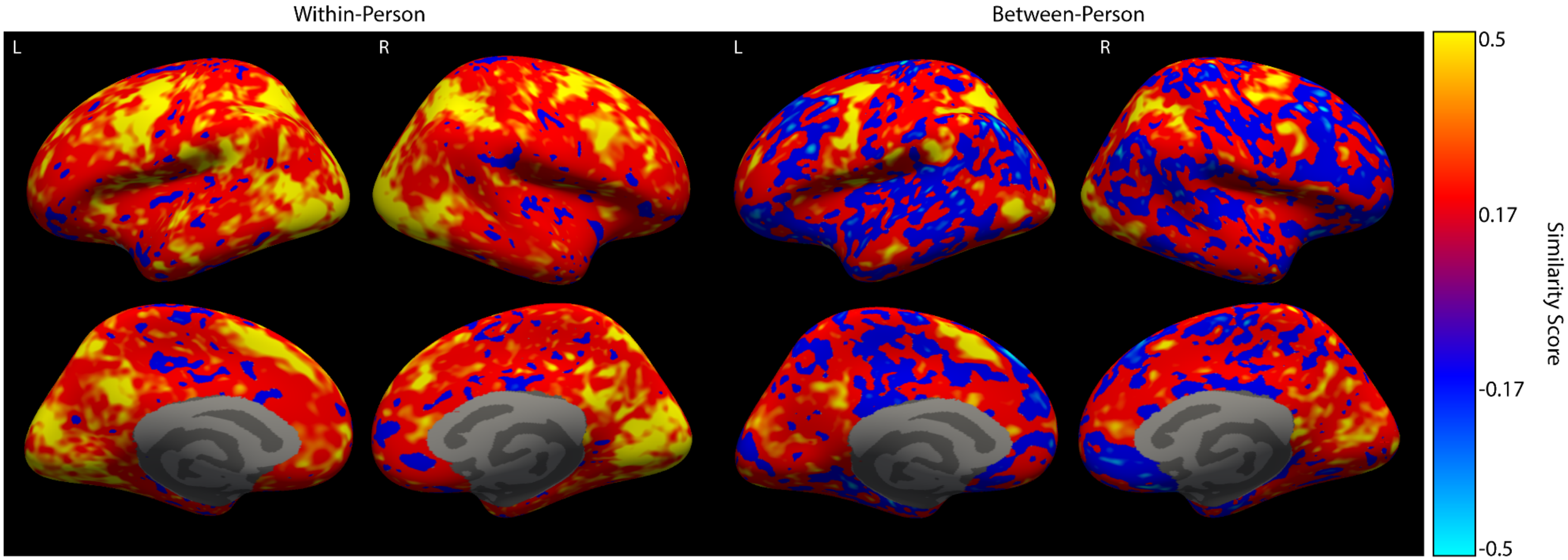

Figure S2: Brain maps of the functional (contrast) similarity scores that were used in the regression model predicting functional similarity from anatomical similarity. Even before controlling for anatomical similarity, within-person functional similarity appears greater than between-person functional similarity. Red and yellow values indicate positive (and high positive) values, whereas blue colors indicate negative values (with light blue representing the lowest negative values). The color scale is consistent across within- and between-person panels. The top row displays the lateral surface, while the bottom row displays the medial surface. Gray medial regions are not included in the cortical surface, and therefore were not included in the analysis.

Figure S3

Person 1 Within-Person Similarity by Task (P1S1-P1S2)

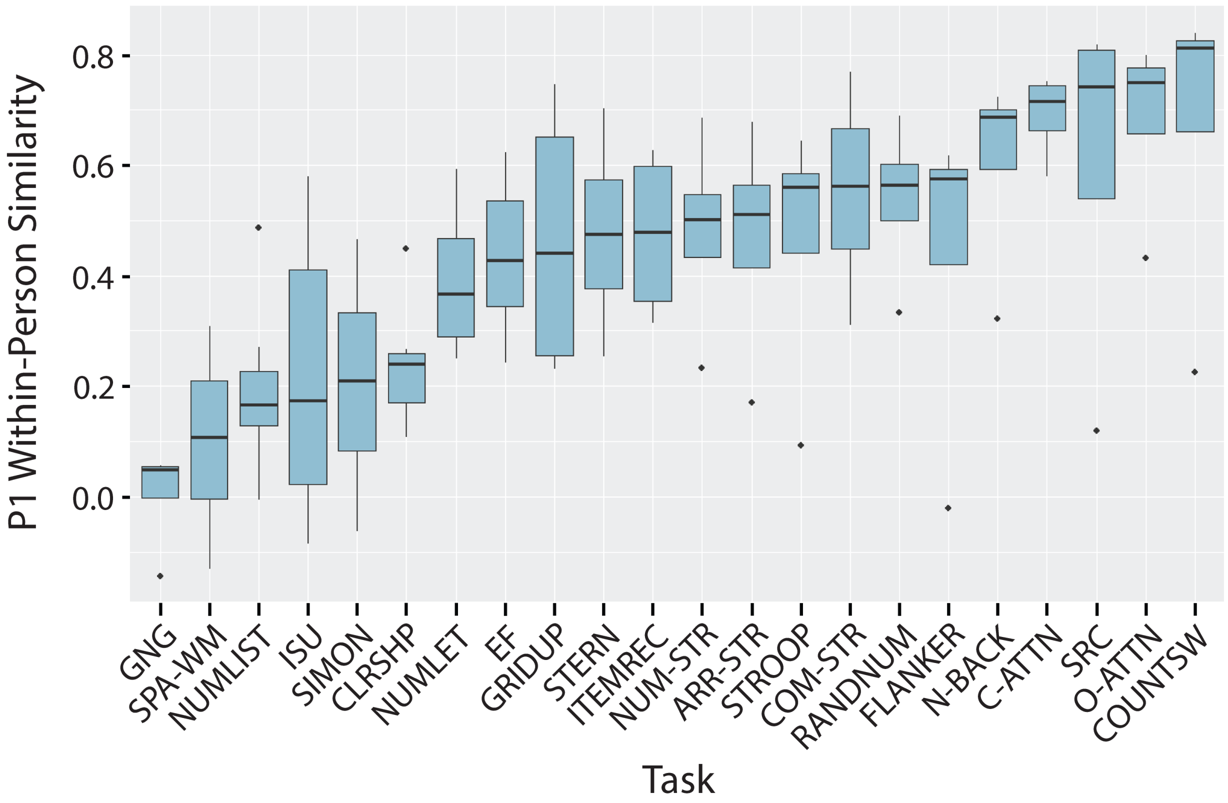

Figure S3: Boxplot displaying within-person similarity scores for only person 1 across the 22 cognitive control tasks (P1S1-P1S2), sorted from lowest (left) to highest (right). The box midline indicates the median, while box edges define the interquartile range. Solid lines reflect 1.5x the interquartile range, and values beyond those are indicated with solid dots. GNG = Go/No-Go, SPA-WM = Spatial Working Memory, NUMLIST = Number-List, ISU = Inhibit, Switch, Update, CLRSHP = Color-Shape, NUMLET = Number-Letter, EF = Executive Function, GRIDUP = Grid Updating, STERN = Sternberg, ITEMREC = Item Recognition, NUM-STR = Number Stroop, ARR-STR = Arrow Stroop, COM-STR = Combined Stroop, RANDNUM = Random Number Generation, N-BACK = Letter *N*-back, C-ATTN = Covert Attention, SRC = Stimulus-Response Compatibility, O-ATTN = Overt Attention, COUNTSW = Counter Switching.

Figure S4

Person 2 Within-Person Similarity by Task (P2S1-P2S2)

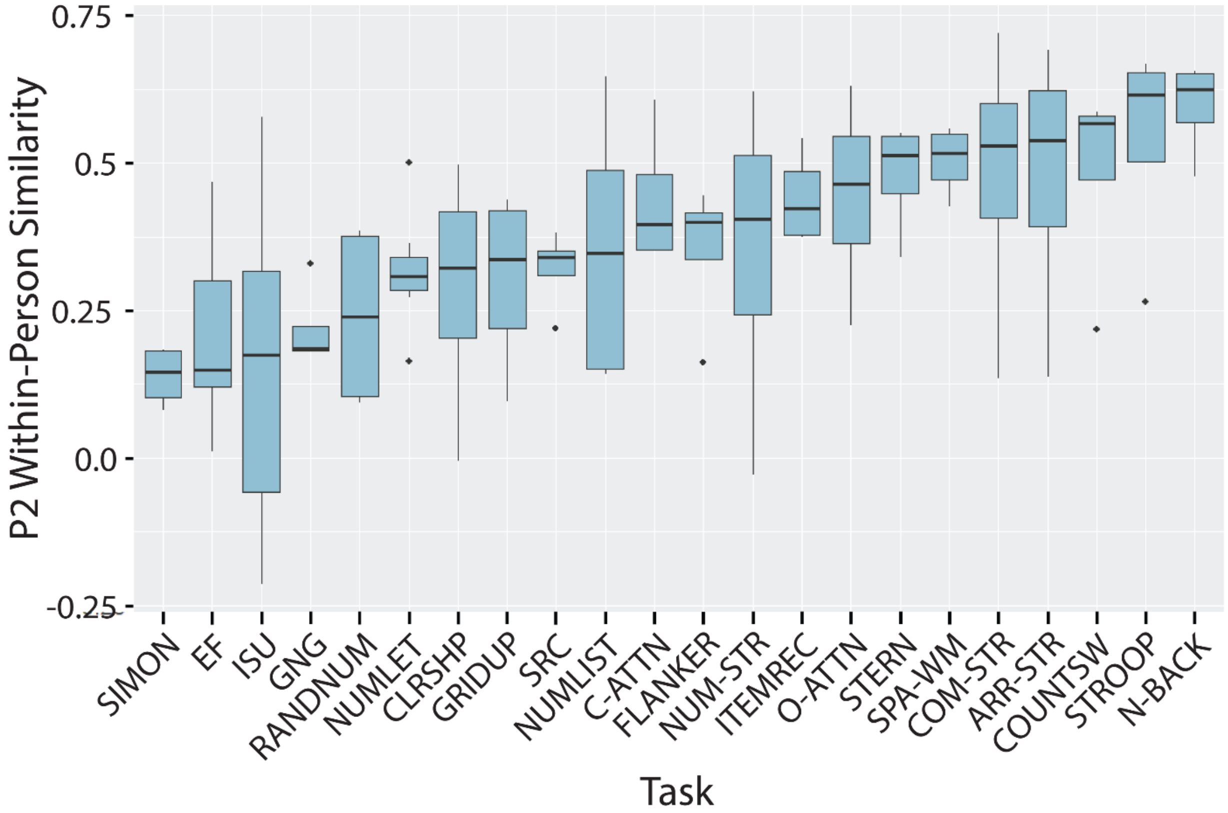

Figure S4: Boxplot displaying within-person similarity scores for only person 2 across the 22 cognitive control tasks (P2S1-P2S2), sorted from lowest (left) to highest (right). The box midline indicates the median, while box edges define the interquartile range. Solid lines reflect 1.5x the interquartile range, and values beyond those are indicated with solid dots. EF = Executive Function, ISU = Inhibit, Switch, Update, GNG = Go/No-Go, RANDNUM = Random Number Generation, NUMLET = Number-Letter, CLRSHP = Color-Shape, GRIDUP = Grid Updating, SRC = Stimulus-Response Compatibility, NUMLIST = Number-List, C-ATTN = Covert Attention, NUM-STR = Number Stroop, ITEMREC = Item Recognition, O-ATTN = Overt Attention, STERN = Sternberg, SPA-WM = Spatial Working Memory, COM-STR = Combined Stroop, ARR-STR = Arrow Stroop, COUNTSW = Counter Switching, N-BACK = Letter *N*-back.

Figure S5

Set 1 Between-Person Similarity by Task (P1S1-P2S1)

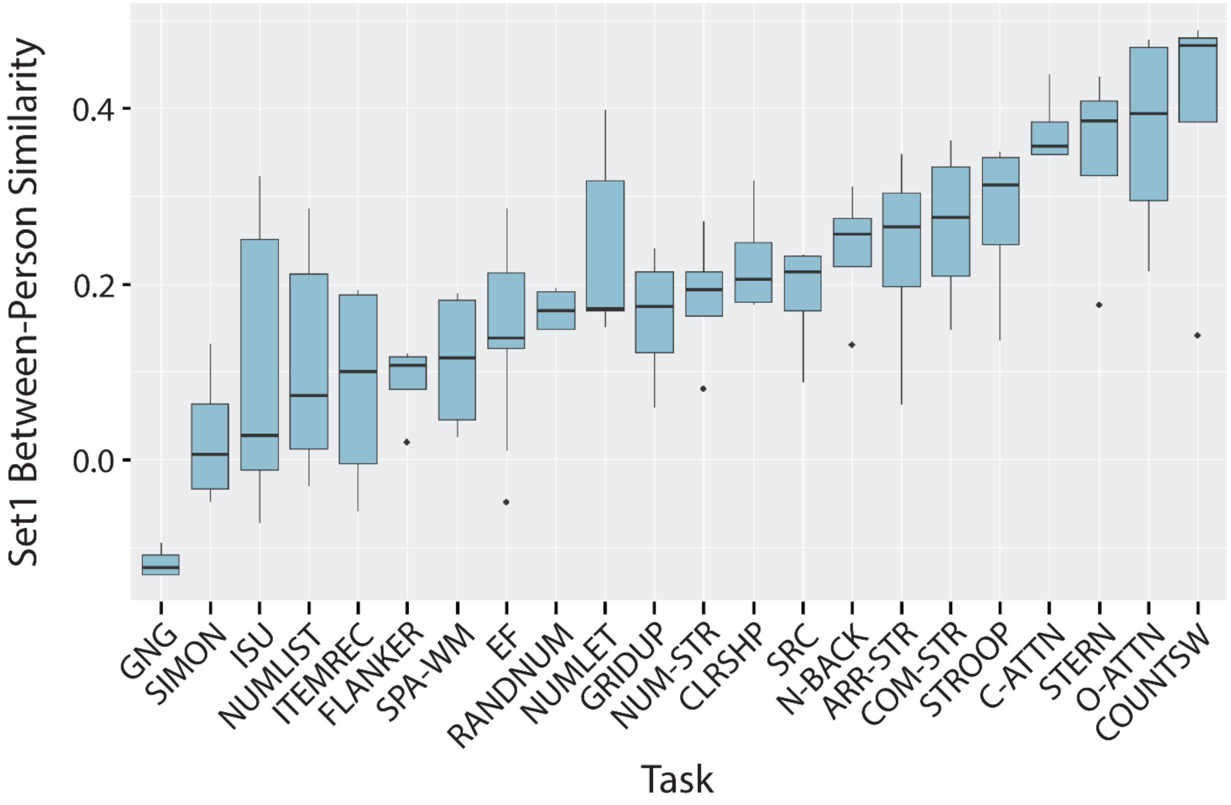

Figure S5: Boxplot displaying between-person similarity scores for only Set1 datasets across the 22 cognitive control tasks (P1S1-P2S1), sorted from lowest (left) to highest (right). The box midline indicates the median, while box edges define the interquartile range. Solid lines reflect 1.5x the interquartile range, and values beyond those are indicated with solid dots. GNG = Go/No-Go, ISU = Inhibit, Switch, Update, NUMLIST = Number-List, ITEMREC = Item Recognition, SPA-WM = Spatial Working Memory, EF = Executive Function, RANDNUM = Random Number Generation, NUMLET = Number-Letter, GRIDUP = Grid Updating, NUM-STR = Number Stroop, CLRSHP = Color-Shape, SRC = Stimulus-Response Compatibility, N-BACK = Letter *N*-back, ARR-STR = Arrow Stroop, COM-STR = Combined Stroop, C-ATTN = Covert Attention, STERN = Sternberg, O-ATTN = Overt Attention, COUNTSW = Counter Switching.

Figure S6

Set 2 Between-Person Similarity by Task (P1S2-P2S2)

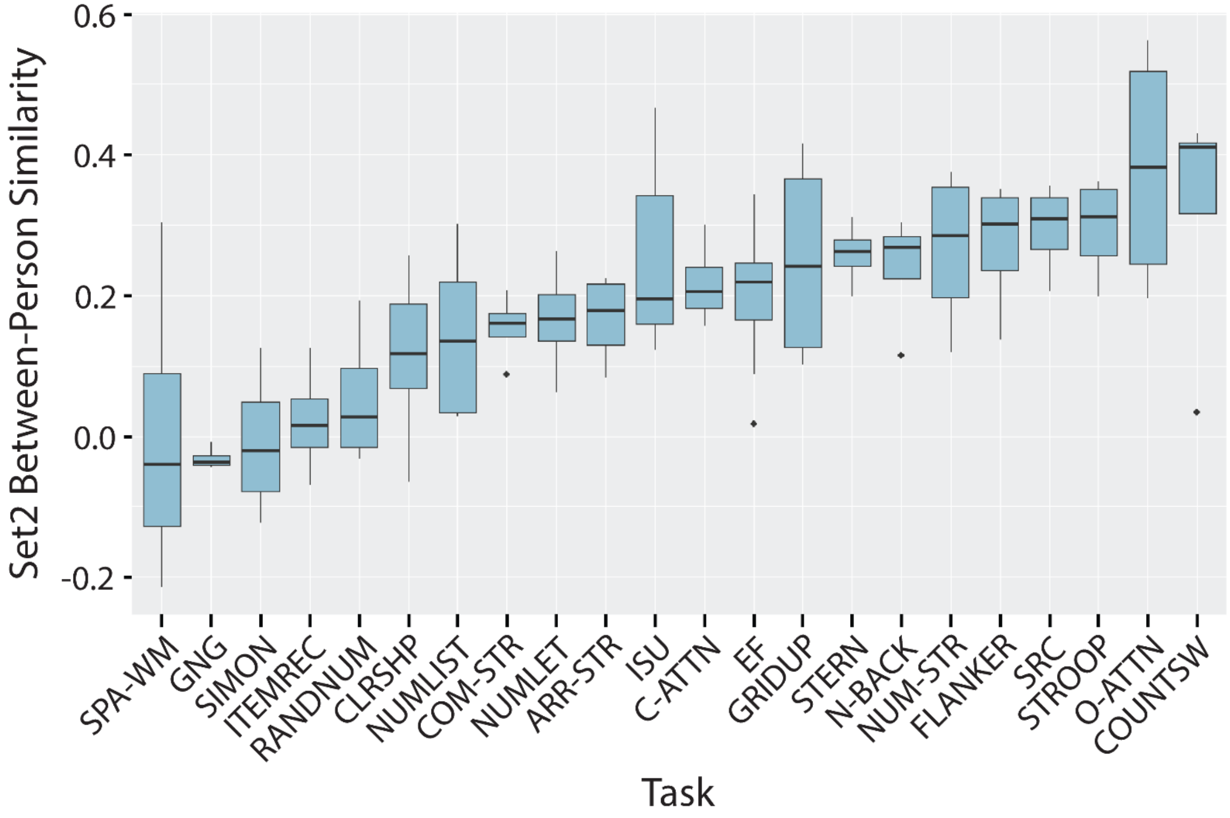

Figure S6: Boxplot displaying between-person similarity scores for only Set2 datasets across the 22 cognitive control tasks (P1S2-P2S2), sorted from lowest (left) to highest (right). The box midline indicates the median, while box edges define the interquartile range. Solid lines reflect 1.5x the interquartile range, and values beyond those are indicated with solid dots. SPA-WM = Spatial Working Memory, GNG = Go/No-Go, ITEMREC = Item Recognition, RANDNUM = Random Number Generation, CLRSHP = Color-Shape, NUMLIST = Number-List, COM-STR = Combined Stroop, NUMLET = Number-Letter, ARR-STR = Arrow Stroop, ISU = Inhibit, Switch, Update, C-ATTN = Covert Attention, EF = Executive Function, GRIDUP = Grid Updating, STERN = Sternberg, N-BACK = Letter *N*-back, NUM-STR = Number Stroop, SRC = Stimulus-Response Compatibility, O-ATTN = Overt Attention, COUNTSW = Counter Switching.
